# Medical Items

**Published:** 1893-08

**Authors:** 


### MEDICAL ITEMS.

Dr. H. E. Felton, son of Rev. W. H. Felton, of Cartersville, has located in Atlanta.

A certain Chicago clergyman announced from the pulpit that "Our dear sister, Mrs. X, is suffering from a serious and painful illness. She is being cared for by our dear brother, Dr. G. Let us all pray for her safety."

The next annual class for instruction in orificial surgery will assemble in Chicago on the morning of September 4th. It will have a four hour daily session during the week. For particulars address Dr. E. H. Pratt, R. 56, Central Music Hall, Chicago.

Dr. John B. Hamilton, of Chicago, has been elected editor of the *Journal of the American Medical Association.* He has been editor of the *Journal* before, and a trustee of the same for several years. Dr Culberson, the former editor, will return to Cincinnati.

The fifth annual meeting of the Tri-State Medical Society of Georgia, Alabama and Tennessee will be held in Chattanooga, October 17th, 18th and 19th. Members of the society and others who wish to present papers should communicate with Dr. Frank Trester Smith, Secretary, Chattanooga, Tenn.

Dr. W. H. Inghram, formerly of Atlanta, has decided to locate in Macon, where he will practice diseases of the eye, ear, nose and throat. We hope Dr. Inghram will succeed in his new home, and we take pleasure in commending him to the good graces of the Macon profession.

IN Madrid, Spain, there is surely the most unique operating theatre in the world. In their wild endeavor to keep out the deadly microbe, those who constructed it provided an immense glass cabinet in which the patient, the operator and assistants and instruments are confined. The spectators witness the operation from the outside. This is nothing but "transcendental antisepticism" and monumental nonsense. At present, antiseptic precautions seem to be heading towards simpler methods instead of the more complicated.

---

Messrs. Parke, Davis & Co. and the Columbia Chemical Co. occupy advertising space which they have contracted for in THE JOURNAL.

We disclaim all responsibility for what either may say in their respective spaces, as that space is theirs to use as they choose, provided the matter inserted does not transcend the bounds set by legitimate medical journalism.

THE ATLANTA MEDICAL AND SURGICAL JOURNAL.

---

The nineteenth annual meeting of the Mississippi Valley Medical Association will occur in Indianapolis, Wednesday, Thursday and Friday, October 4, 5 and 6, 1893.

A general session will be held each morning, and the afternoons will be devoted to section work. There will be three sections at this meeting, viz.: One on General Medicine, one on General Surgery and one on Obstetrics and Gynecology; the last mentioned having been added since the last meeting.

The indications at present are, that for genuine scientific work, this will be one of the best meetings in the history of the association. The attendance will probably be unusually large, as many physicians expect to make their visit to the World's Fair at this time. Chicago is but a few hours' ride from Indianapolis, and there is no more delightful time of the year in which to visit the World's Fair than this. Holders of tickets to Chicago on any line passing through Indianapolis will be entitled to stop-over privileges at the latter point. Cheap rates will also prevail between these two cities.

The profession of Indianapolis is united in extending a cordial invitation to physicians and their families to attend the meeting.

Reduced railroad rates will be provided, further notice of which will be given.

The Secretary will be glad to receive titles from those physicians desiring to favor the Association with papers. It is especially requested that these titles be sent as early as possible, in order to give ample opportunity for the appointment of leaders in discussion.

The Secretary will take pleasure in giving any information in connection with the meeting.

FREDERICK C. WOODBURN, Secretary.
No. 399 College Avenue.

